# Bo-Gan-Whan regulates proliferation and migration of vascular smooth muscle cells

**DOI:** 10.1186/s12906-016-1292-9

**Published:** 2016-08-22

**Authors:** Kang Pa Lee, Jai-Eun Kim, Hyuck Kim, Hae Ryong Chang, Dae Won Lee, Won-Hwan Park

**Affiliations:** 1 Department of Bio-Science, College of Natural Science, Dongguk University, Dongdae-ro 123, Gyeongju, Gyeongsangbuk-do 38066 Republic of Korea; 2 Department of Pathology, College of Korean Medicine, Dongguk University, Dongguk-Ro 32, Goyang, Gyeonggi-do 10326 Republic of Korea; 3 Department of Diagnosis, College of Korean Medicine, Dongguk University, Dongguk-Ro 32, Goyang, Gyeonggi-do 10326 Republic of Korea

**Keywords:** Bo-Gan-Whan, Vascular smooth muscle cells, Platelet-derived growth factor-BB, Extracellular signal-regulated kinase 1/2, p38 mitogen-activated protein kinase

## Abstract

**Background:**

Bo-Gan-Whan (BGH), a Korean polyherbal medicine, is used as a hepatoprotective drug. It has six natural sources, and has been demonstrated to have anti-oxidative, anti-cancer, and anti-inflammatory properties; however, its effect on vascular diseases remains unclear.

**Methods:**

Cell viability and proliferation assays were employed using an EZ-Cytox Cell Viability Assay Kit. Platelet-derived growth factor (PDGF)-BB-induced vascular smooth muscle cell (VSMC) migration was measured by scratch wound healing assay and Boyden chamber assay. The expression levels of the phosphorylated signaling proteins relevant to proliferation, including extracellular signal-regulated kinase (ERK) 1/2 and p38 mitogen-activated protein kinase (MAPK) were determined by western blot analysis. Chromatogram and mass analysis were employed by Ultra Performance Liquid Chromatography (UPLC) system. Cell prolife ration and migration were also explored using the PDGF-BB-induced aortic sprout assay.

**Results:**

BGH (100–500 μg/mL) significantly inhibited the proliferation and migration of PDGF-BB-stimulated VSMCs through the reduced phosphorylation of ERK1/2 and p38 MAPK in comparison to untreated PDGF-BB-stimulated VSMC. Moreover, we identified the paeoniflorin as the major composition of BGH.

**Conclusions:**

We suggest that BGH may have an anti-atherosclerosis effect by inhibiting the proliferation and migration of PDGF-BB-stimulated VSMCs through down-regulation of ERK1/2 and p38 MAPK phosphorylation.

## Background

Vascular disorders are among the major causes of health problems or death, particularly in western countries [1]. Acute coronary diseases are associated with mortality and therefore require urgent medical attention such as stent therapy to restore blood flow to a narrowed blood vessel [2]. However, the stent therapy has an inevitable risk factor such as vascular restenosis [3]. Particularly in vascular lesion, abnormal physiological responses of smooth muscle cells (VSMCs) underlying cell migration and proliferation are the major mechanism of developing vascular restenosis [4]. Therefore, controlling pathological progressions of VSMCs is one of the major methods under consideration to prevent the restenosis [5, 6].

Several studies have shown that VSMCs motility and hyperplasia in response to arterial pathogenesis were stimulated by platelet-derived growth factor (PDGF) [7, 8]. PDGF-BB can stimulate arterial pathogenesis signal cascades such as PDGF beta receptor and its downstream signaling molecules, consequently resulted in an increasing phosphorylated p38 mitogen-activated protein kinase and activated of extracellular signal-regulated kinase 1/2 [9, 10].

Recently, functional foods or medical food supplements have been used in health-promotion or disease prevention-strategies. This requires the discovery and development of products from natural sources that may provide alternative interventions to currently approved medicine [11, 12]. The majority of patients who undergo angioplasty through stents, require inevitable medication for a long period of time to avoid the progression or reoccurrence of restenosis [13]. For these reasons, Korean traditional medicines have been used as an alternative to treat diverse human diseases and to maintain good health [14]. Thus, in this study, we investigated the efficacy of Bo-Gan-Whan (BGH) in restenosis. According to the Korean medical encyclopedia, BGH has been traditionally prescribed for infirmed liver and for general hepatic-protection. However, to date, the anti-migration and proliferation of VSMCs after treatment with BGH is not fully understood. Hence, we attempted to investigate the anti-restenosis effect of BGH on PDGF-BB-stimulated VSMCs to provide fundamental data for alternative medicine development.

## Methods

### Preparing reagents

The EZ-Cytox Cell Viability Assay Kit were purchased from Daeil Lab Service (Seoul, Korea) and cell culture materials were purchased from Gibco-BRL (Gaithersburg, MD, USA), respectively. Recombinant PDGF-BB was obtained from R&D systems (Minneapolis, MN, USA). Specific antibodies for GAPDH, ERK1/2, phosphorylation of ERK1/2, p38, and phosphorylation of p38 for analysis of western blots, were purchased from Santa Cruz Biotechnology (Santa Cruz, CA, USA). All other chemicals were purchased from Sigma (St. Louis, MO, USA).

### Plant material and extraction

BGH (5 g *Ostericum koreanum rhizome*, 5 g *Anglicae gigas* root, 5 *g Ledebouriella seseloides* root, 5 g *Paeonia lactiflora* root, 5 g *Rehmannia glutinosa* root, and 5 g *Cnidium officinale*. Makino) was obtained from Dongguk University Oriental Hospital, Korea which has been prepared specifically for this study from the commercially-available products. The plants were blended (a total of 30 g), and the crude powder was boiled in 1000 mL of sterile deionized water at 100 °C for 3 h. The aqueous extracts were concentrated and evaporated at 60 °C under vacuum. The extract was then lyophilized by freeze-drying at −60 °C.

### Isolation and cultivation of primary VSMCs from rats

Aortic VSMCs were isolated from male Sprague–Dawley (SD) rats following previously described protocols [15]. VSMCs were cultured in Dulbecco’s Modified Eagle’s Medium (DMEM) low glucose supplemented with 10 % fetal bovine serum (FBS), 100 U/mL penicillin, 100 g/mL streptomycin, and 200 mM glutamine at 37 °C under a humidified 95 % air/5 % CO_2_ mixture (v/v). All protocols for animal experiments were approved by the ethics committee of Dongguk University (IACUC-2014-009).

### The cell viability assay

VSMCs were seeded at 2 × 10^4^ cells/mL in a 96-well microplate containing DMEM and incubated for 24 h. The cells were then incubated with different concentrations of BGH (10–500 μg/mL) in DMEM for 24 h. The cell viability of VSMCs was determined by the EZ-Cytox Cell Viability Assay Kit at 450 nm. The effects of BGH were then determined based on the relative cell viability of the treated group in comparison to the untreated group.

### The assay of VSMCs’ proliferation

VSMCs (2 × 10^4^ cells/mL) were seeded in a 96-well microplate containing DMEM and incubated for 24 h. These cells were incubated in serum-free media for 12 h. VSMCs were then incubated with different concentrations of BGH (10–500 μg/mL) and PDFG-BB (10 ng/mL) for an additional 24 h. The proliferation rate of VSMCs was determined by the EZ-Cytox Cell Viability Assay Kit at 450 nm.

### Scratch wound healing assay

VSMCs (4 × 10^4^ cells/mL) were incubated in a 6-well dish in growth medium for 24 h and then incubated in serum-deprivation media for 24 h. To induce a migrating zone in transverse scratch wound, each monolayer of VSMCs was scratched with a sterilized 200 μL-tip. Subsequently, VSMCs were treated with PDGF-BB (10 ng/mL) and BGH (100–500 μg/mL) for an additional 24 h. VSMC morphological changes were examined and recorded using an inverted microscope and CCD camera (IX71; Olympus, Tokyo, Japan). The migrating zone was examined and analyzed between 0 h to 24 using Image J software.

### Boyden chamber assay

The PDGF-BB-mediated VSMC migration assay was performed using a Boyden chamber, as previously described [15]. VSMCs were harvested from the grown-up in serum-free media for 24 h. A density of 1 × 10^6^ cell/mL of VSMCs in 50 μL DMEM supplemented 0.1 % bovine serum albumin (BSA) in the upper chamber, which was then inserted in the lower chamber containing PDGF-BB (10 ng/mL) and various concentrations of BGH (100–500 μg/mL) in 28 μL DMEM supplemented 0.1 % BSA. The migrated VSMCs on the membrane for 90 min were analyzed using Image J software.

### Western blot analysis

VSMC lysates were separated by electrophoresis using 12 % acrylamide gels and then transferred to polyvinylidene difluoride membranes in transfer buffer at 4 °C for 2 h. The membrane was treated with Tris-buffered saline containing 5 % BSA at room temperature for 1 h and then incubated overnight at 4 °C with antibodies for phosphorylated ERK1/2 (p-ERK1/2) or p38 (p-p38), total ERK1/2 (T-ERK1/2) or p38 (Tp38), and GAPDH at 1:1000 dilution. The membranes were washed in Tris-buffered saline containing 0.1 % Tween 20, and then incubated with a 1:1000 dilution of anti-IgG secondary antibody conjugated to horseradish peroxidase for 1 h. The expression levels of each protein were analyzed via chemiluminescence and quantified using Image J Software.

### The assay of aortic sprout ring growth

Ex vivo properties of vessels were measured by an aortic sprout assay, as previously described [16]. Owing to the protocol, the endothelium and adventitium of the aorta from SD rats (5-weeks-old, *n* = 4) were removed mechanically and enzymatically, and the internal vessels were cut into 1-mm^2^ strips. The strips were embedded in 48-well plates coated with matrigel, and then treated with PDGF-BB (10 ng/mL) and BGH (100–500 μg/mL) in FBS-free DMEM for five days. The strips were stained with Diff-Quik (Baxter Healthcare) and photographed, and the length of sprout was analyzed using Image J software.

### Ultra -performance liquid chromatography (UPLC)-based analysis

UPLC-based fingerprinting was performed with an UPLC system (Waters, Corporation, Milford, USA), equipped with a quaternary pump, a vacuum degasser, diode-array detector and Waters software. Separation was performed using a UPLC™ BEH C_18_ column (1.7 μm, 2.1 mm × 50 mm). The mobile phase was a mixture of 0.1 % (v/v) of acetonitrile and water containing 0.1 % (v/v) formic acid at a flow rate of 0.3 mL/min. A standard solution containing paeoniflorin (Sigma-Aldrich, MO, USA) was prepared by dissolving these compounds in distilled water (10 mg/100 mL). The solution was filtered through a 0.45 μm membrane filter, after which UPLC was performed.

### Statistical analysis

GraphPad Prism (GraphPad, San Diego, USA) was used to analyze statistical data analysis. The results were presented as the mean ± standard error (SE) of at least three independent experiments (*n* ≥ 3). The results were assessed using a Student’s t-test and one-way ANOVA followed by Tukey's multiple range test. Statistical significance was considered at *P* < 0.05.

## Results

### BGH inhibits the proliferation of PDGF-BB-induced VSMCs

**Fig. 1:**
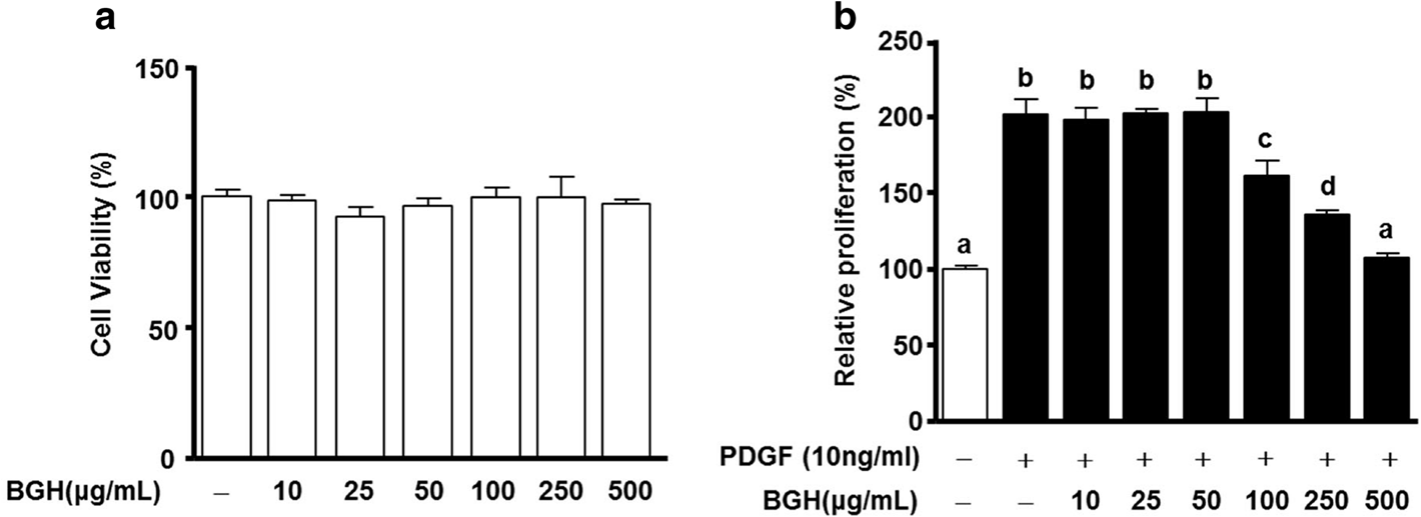
The effect of Bo-Gan-Whan (BGH) on proliferation of PDGF-BB-stimulated VSMCs. **a** Cell Viability and **b** Proliferation Rates of VMSCs after 24 h treatment with BGH in a dose-dependent manner (10, 25, 50, 100, 250, and 500 μg/ mL). The graph shows the representative cell viability from three independent experiments. Data are presented as mean ± standard error. Values with the same superscript letter are not significantly different as analyzed by Tukey’s multiple range test (*P* < 0.05)

To test the effect of BGH on PDGF-BB-induced proliferation of VSMCs, a 2,3-bis [2-methyloxy-4-nitro-5-sulfophenyl]-2H-tetrazolium-5-carboxanilide (XTT) assay was employed using EZ-Cytox Cell Viability Assay Kit. The data demonstrated that BGH did not induce altered cell viability and morphology at defined concentrations up to 500 μg/mL compared with the untreated group (*n* = 3, Fig. 1a). Treatment with BGH at varying concentrations between 100–500 μg/mL inhibited PDGF-BB (10 ng/mL)-induced proliferation of VSMCs in a dose-dependent manner. The optimum effect was observed at 500 μg/mL of BGH (*n* = 3, Fig. 1b).

### BGH inhibits the migration of PDGF-BB-stimulated VSMCs

**Fig. 2:**
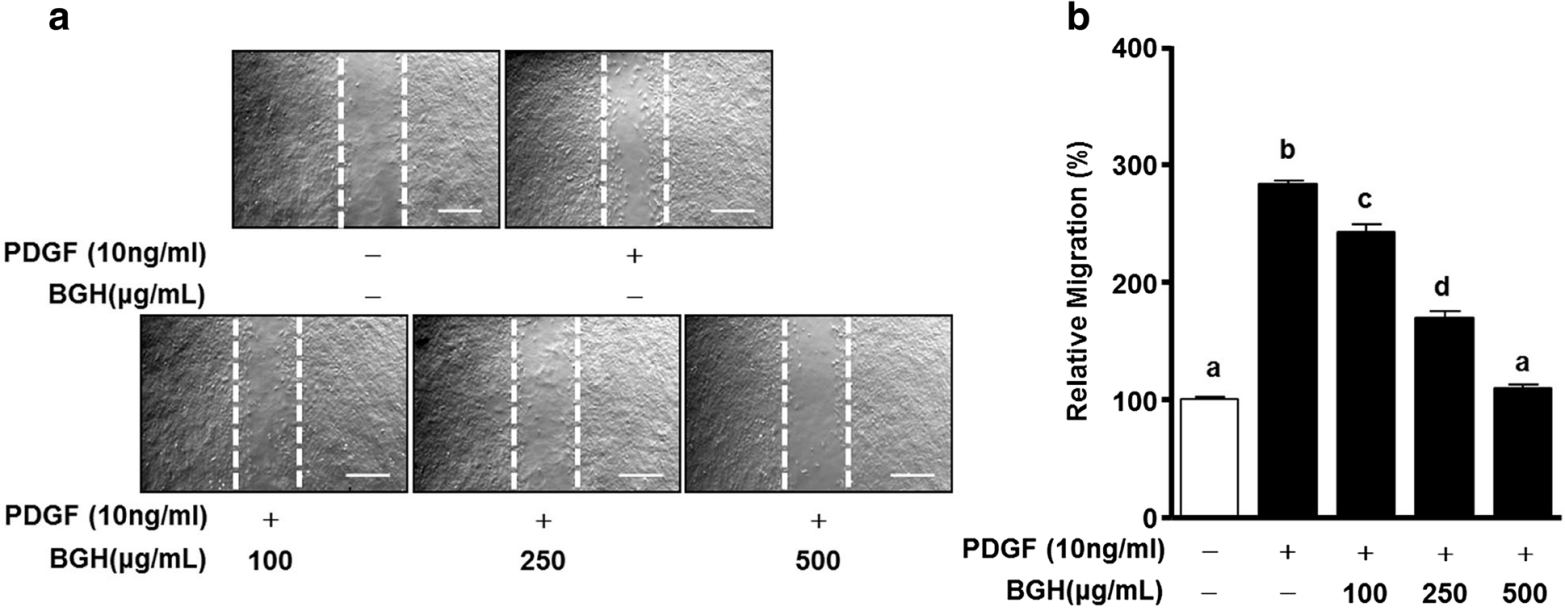
Effects of Bo-Gan-Whan (BGH) on scratch wound-healing of PDGF-BB-stimulated VSMCs. VSMCs were treated with BGH (100–500 μg/mL) and PDGF-BB (10 ng/mL) for 24 h. **a** *Dotted white lines* indicate the initial scratches. Magnification, 100×. Bar = 500 μm. **b** Migration rates of VMSCs relative to the untreated group (%). Results are presented as mean ± standard error. Values with the same superscript letter are not significantly different as analyzed by Tukey’s multiple range test (*P* < 0.05)

**Fig. 3:**
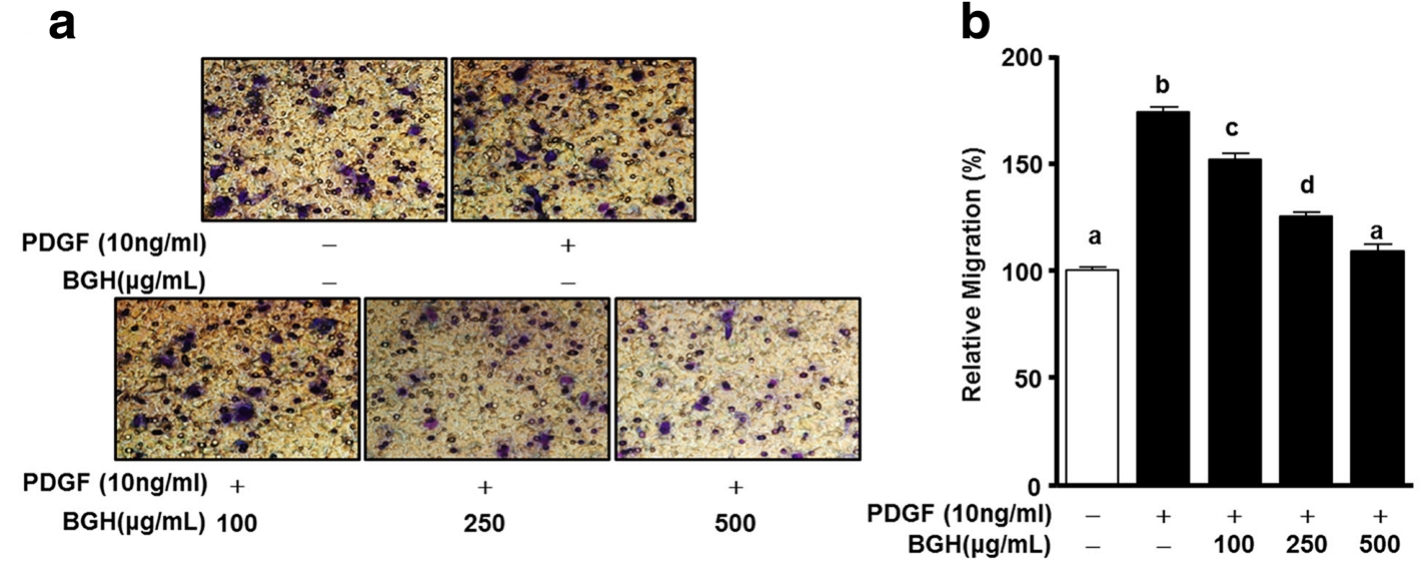
Effects of Bo-Gan-Whan (BGH) on migration of PDGF-BB-stimulated VSMCs. VSMCs were treated with BGH (100, 250, and 500 μg/ mL) and PDGF-BB (10 ng/mL) for 90 min. **a** Microphotographs of the migration patterns of VSMCs on membranes. The spots are Diff quick-stained cells. Magnification = 200×. **b** Migration rates of BGH and PDGF-BB-treated VMSCs relative to the untreated group (%). Results are presented as mean ± standard error. Values with the same superscript letter are not significantly different as analyzed by Tukey’s multiple range test (*P* < 0.05)

One of the causes of atherosclerosis is an abnormal migration of VSMCs in pathogenic condition that be induced by PDGF and cytokines [7, 8]. Therefore, we determined the measuring anti-migration effect of BGH by the scratch wound healing and the boyden chamber assay with PDGF-BB stimulation. First, in the case of scratch wound healing assay, BGH significantly inhibited the PDGF-BB-stimulated migration of VSMCs as shown by the reduction of the scratch area, in a dose-dependent manner (Fig. 2). The data confirms that the maximum inhibitory effect of BGH treatment on PDGF-BB (10 ng/mL)-induced cell migration was achieved at 500 μg/mL (*n* = 3, Fig. 2). Next, the boyden chamber assay was performed to assess whether BGH could inhibit cell migration of the PDGF-BB-stimulated VSMCs within a short period. VSMCs were treated with BGH (100, 250, and 500 μg/ mL) and PDGF-BB (10 ng/ mL) for 90 min. As shown in Fig. 3, PDGF-BB (10 ng/ml) increased VSMC migration, whereas BGH (100, 250, and 500 μg/ mL) significantly inhibited PDGF-BB (10 ng/ml)-induced migration in a dose-dependent manner (*n* = 6, Fig. 3).

### BGH reduces the phosphorylation of p38 MAPK and ERK1/2 in PDGF-stimulated VSMCs

**Fig. 4:**
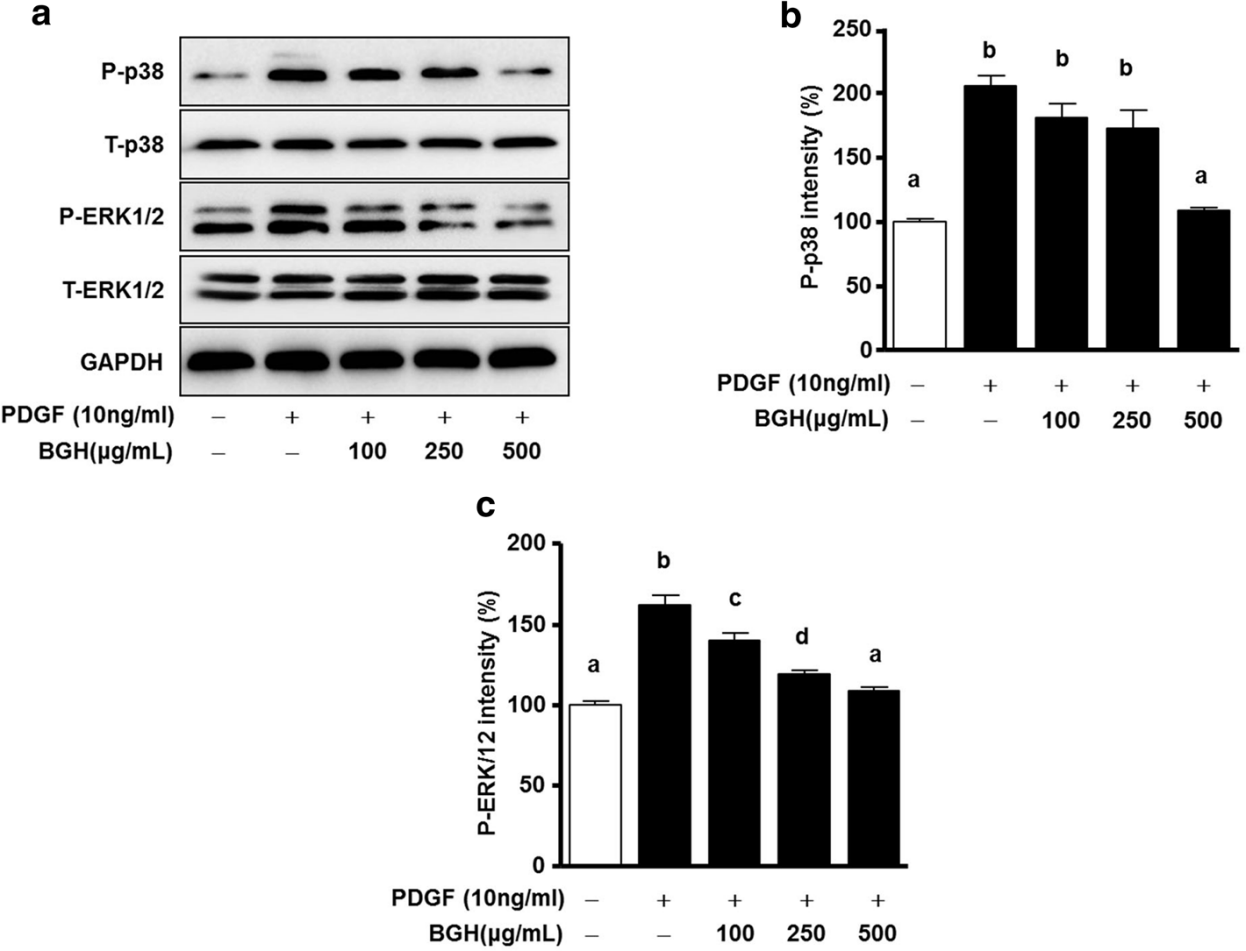
Effect of Bo-Gan-Whan (BGH) on PDGF-BB induced phosphorylation of ERK1/2 and p38 MAPK in VSMCs. VSMCs were pretreated with or without (100, 250, and 500 μg/ mL) BGH for 1 h and then stimulated with 10 ng/mL of PDGF-BB for 15 min. For statistical analysis, densitometry of the band representing the phosphorylated form (p-ERK1/2 and p-p38) was normalized to the expression of their respective total forms (T-ERK1/2 and T-p38). Western blot analysis of phosphorylated ERK1/2 (P-ERK1/2) and p38 MAPK (p-p38) (**a**) and their corresponding relative intensities (**b**) and (**c**). Results are presented as the mean ± standard error of three independent experiments. Values with the same superscript letters are not significantly different as analyzed by Tukey’s multiple range test (*P* < 0.05)

Several studies have suggested that a key signal cascade in PDGF-BB-induced VSMCSs is p38 MAPK and ERK1/2 [10, 15]. Therefore, we tested whether BGH inhibits the expression of phosphorylated p38 MAPK and ERK1/2 in PDGF-BB-induced VSMCs using western blot analysis. PDGF-BB (10 ng/mL) increased the phosphorylation of ERK1/2 and p38 MAPK in VSMCs (*n* = 4, Fig. 4). The phosphorylation of p38 MAPK was only reduced at a concentration of 500 μg/mL BGH (Fig. 4b) whereas the phosphorylation of ERK1/2 was inhibited by BGH treatment (100, 250, and 500 μg/ mL) in a dose-dependent manner (Fig. 4c).

### BGH inhibits PDGF-BB-stimulated sprout outgrowth

**Fig. 5:**
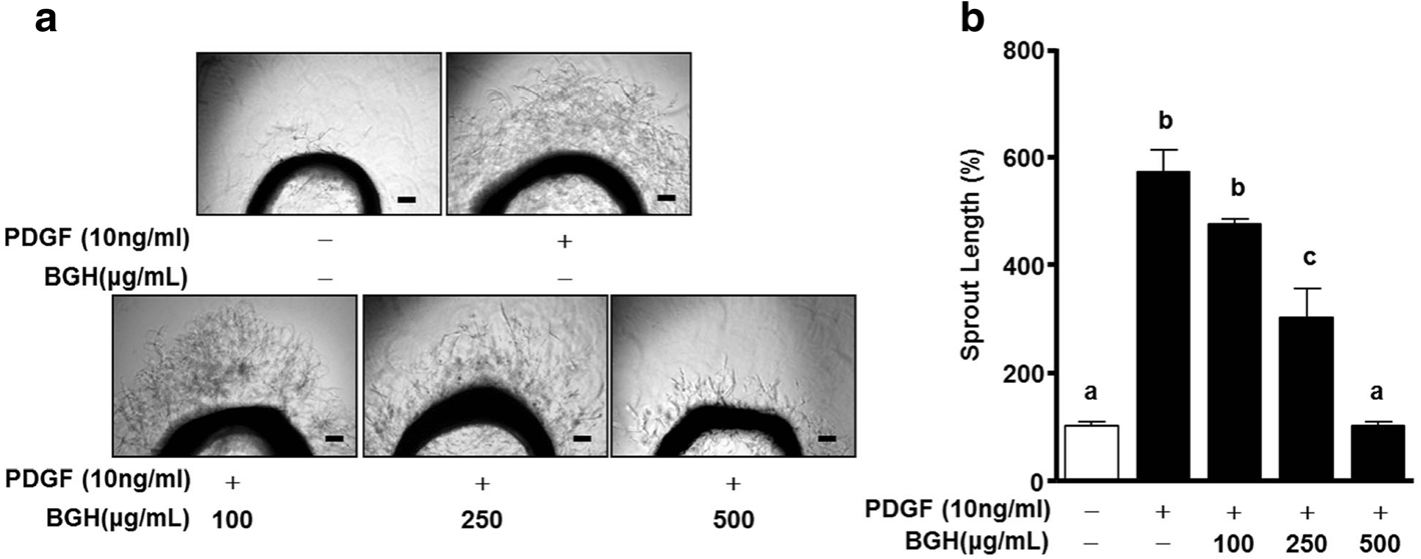
Effect of Bo-Gan-Whan (BGH) on PDGF-BB-induced aortic sprout growth. The microphotographs of the aortic rings embedded in Matrigel, treated with or without BGH (100, 250, and 500 μg/ mL) and PDGF-BB (10 ng/ml) are shown (**a**). The results were observed after four days of incubation. **b** Also shown is the sprout outgrowth level in the untreated and BGH- and PDGF-BB-treated aortic rings, expressed as 100 % (n = 3). Results are presented as the mean ± standard error of three independent experiments. Values with the same superscript letters are not significantly different as analyzed by Tukey’s multiple range test (*P* < 0.05)

To confirm the migration and proliferation of VSMCs ex vivo, the aortic sprout outgrowth assay was performed. PDGF-BB significantly increased the sprout outgrowth from the aortic strips, whereas varying concentrations of BGH (100, 250, and 500 μg/ mL) induced elimination of the PDGF-BB-induced outgrowths in a dose-dependent manner. This inhibitory response reached a maximum level at 500 μg/mL of BGH (*n* = 3; Fig. 5).

### The analysis of chromatogram and mass in the composition of BGH

**Fig. 6:**
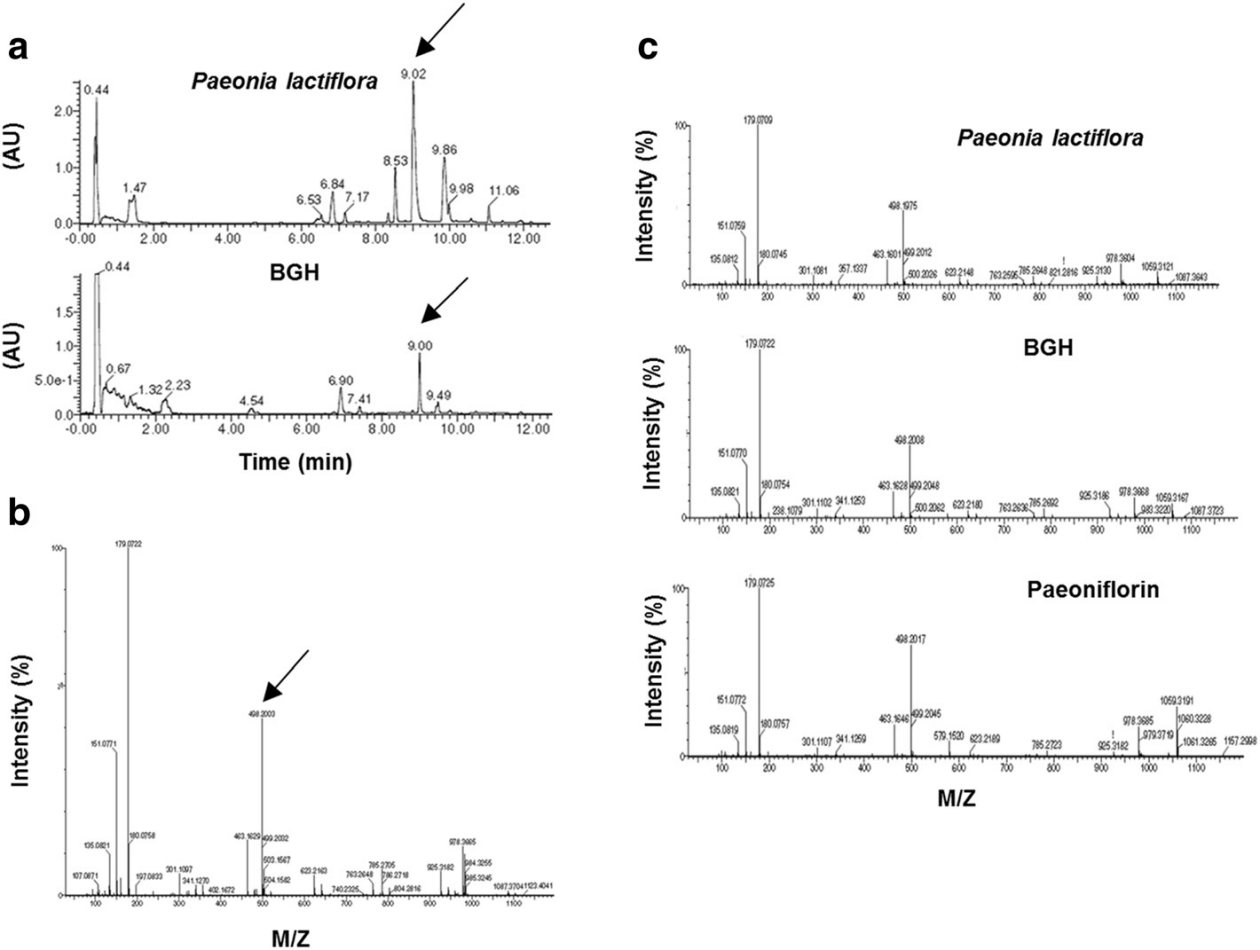
UPLC-based Mass (MS) analysis of Bo-Gan-Whan (BGH). **a** The chromatograms of *Paeonia lactiflora* root (*upper* penal) and BGH (*bottom* penal) during 12 min. The *Arrows* indicate the major peak of 9.01 and 9.00 min, respectively. **b** The mass profilie in the chromatogram at 9.0 min. The *arrow* is indicated in 498.2003 M/Z. **c** MS spectra of *Paeonia lactiflora* root, BGH and Paeoniflorin. M/Z: mass to change ratio

As shown in Fig. 6, to identify the composition of BGH, we performed UPLC-based analysis. First, we detected a major peak between *Paeonia lactiflora* root and BGH at approximately 9.00 min employed UV-spectrum (Fig. 6a). Next, we identified paeoniflorin from the mass of constituents in its retention time, which was of 498.2003 molecular weights (Fig. 6b). Finally, we found BGH and paeoniflorin according to MS spectra among *Paeonia lactiflora* root (Fig. 6c).

## Discussion

In this study, we demonstrated that Bo-Gan-Whan (BGH), a Korean polyherbal medicine, has an inhibitory effect on VSMC migration and proliferation in response to PDGF-BB as revealed by the results obtained from the scratch-wound healing and Boyden chamber assay and sprout aortic ring assays, respectively. Moreover, it was demonstrated through western blot analysis that the modulation of MAPKs is the major signal that is activated in the pathogenesis of VSMCs through activation of the ERK1/2 and p38 MAPK pathways. These results were confirmed from the ex vivo analysis of PDGF-BB-induced VSMCs’ migration and proliferation. The data on ex vivo analysis, through outgrowth of vessel sprouts from the aortic strips assay, show that BGH treatment can significantly reduce VSMC migration and proliferation after PDGF-BB stimulation.

Abnormal proliferation of VSMCs is a key to the vascular pathological conditions such as atherosclerosis and restenosis. Moreover, excessive migration of VSMCs in vascular walls is one of the critical causes of vascular neointima formation [4]. Therefore, suppression of VSMC migration and proliferation may be one of the protective strategies against restenosis or neointima formation in atherosclerosis. Specifically, the present study shows that BGH could be a potential medicine or supplement for PDGF-BB-stimulated vascular remodeling since it can remarkably inhibit VSMC proliferation and migration at the highest dosage (500 μg/mL of BGH). Therefore, BGH could be a potential therapeutic agent for preventing or treating pathological processes in restenosis and atherosclerosis. Moreover, the proliferative ability of various cells including VSMCs was associated closely with MAPK phosphorylation [15, 16]. Various studies have shown that MAPKs are important signaling molecules in migration and proliferation in response to PDGF-BB stimulation in VSMCs [7, 8]. In this study, PDGF-BB-induced proliferation and outgrowth of vessel sprouts from the aortic strips were suppressed by BGH treatment. These results imply that BGH exerts an inhibitory effect on VSMC proliferation by inhibiting pathogenic vascular remodeling.

In normal vascular function, endothelial cells prevent abnormal vascular remodeling such as hyperplasia of smooth muscle cells. However, this protective role of endothelial cells is no longer evident in patients following exposure to stents, and they become more prone to a number of risk factors that cause the narrowing of the vascular lumen [17, 18]. Some review articles suggest that administration of drugs and vitamins, either orally or intravenously, is essential to prevent restenosis and in-stent restenosis [19–21]. Although this therapeutic strategy has been previously tested, it has not been shown to be consistently helpful. Therefore, development of functional foods has been gaining increasing attention. For these reasons, the development of standardized and effective alternative medicine is essential [11, 12].

The development of atherosclerosis is strongly linked to lipid- metabolism [13, 17]. In addition to the anti-atherosclerosis therapeutic activity of BGH, it also exhibit hepato-protective effect by reducing liver damage that is strongly associated with the development of atherosclerosis. M. Ishigami et al. reported that apoE inhibits VSMC proliferation [22]. For this reason, we assume that BGH can be among those hepato-protective drug that can be prescribed in alternative in Korea. BGH can be derived from six natural sources namely *Ostericum koreanum* rhizome, *Anglicae gigas* root, *Ledebouriella seseloides* root, *Paeonia lactiflora* root, *Rehmannia glutinosa* root, and *Cnidium officinale*. Makino. Bisabolangelone, one of the active components of *Ostericum koreanum*, has been reported to have an anti-inflammatory effect [23]. The crude extract of *Anglicae gigans* Radix showed anti-tumor activity, anti-inflammation activity, and anti-aggregation of platelets. Moreover, a component of Anglicae gigans, decursin, has been reported to have anti-tumor activity [24–27]. Bergapten, is one of the major active components from Ledebouriella seseloides and has been reported to have an anti-tumor effect [28, 29]. Paeonia Radix is the root of a traditional oriental medicinal herb named Paeonia lactiflora Pallas that has been used to treat liver diseases and has therapeutic effect in rheumatoid arthritis [30]. *Rehmannia glutinosa*, on the other hand, is widely used owing to its pharmacological effect on the blood system, immune system, endocrine system, cardiovascular system, and the nervous system [31]. *Cnidii Rhizoma*, a dried root of *Cnidium officinale* Makino has an anti-cancer effect [32, 33]. The extract form the rhizome of *Cnidium officinale* has been reported to show anti-inflammatory and anti-cancer activities [34]. Above all, the Korean polyherbal medicine, BGH has been used for liver protection. In present study, we identified paeoniflorin as the major component of BGH as revealed by the data using the UPLC. Some studies reported the paeoniflorin have hepato-protective and anti-atherosclerosis effect [35, 36]. Therefore, we suggest that BGH may be used as a functional food for prevention atherosclerosis and restenosis.

## Conclusion

The present study has demonstrated that BGH inhibits cell migration, proliferation, and phosphorylation of MAPKs in response to PDGF-BB stimulation in VSMCs. BGH also attenuated the PDGF-BB-induced aortic sprout outgrowth. From these results, the data suggests that BGH could be a potential and a promising therapeutic agent for the development of anti-restenosis or anti-atherosclerotic drugs.

## Abbreviations

BGH: Bo-Gan-Whan
BSA: Bovine serum albumin
DMEM: Dulbecco’s Modified Eagle’s Medium
ERK: Extracellular signal-regulated kinase
FBS: Fetal bovine serum
MAPK: Mitogen-activated protein kinases
PDGF: Platelet-derived growth factor
UPLC: Ultra Performance Liquid Chromatography
VSMC: Vascular smooth muscle cell
